# *EGFR*突变影响非小细胞肺癌PD-L1表达的研究进展

**DOI:** 10.3779/j.issn.1009-3419.2019.12.08

**Published:** 2019-12-20

**Authors:** 晓玉 智, 卫威 李, 少伟 王, 进良 汪

**Affiliations:** 1 100853 北京，解放军总医院第一医学中心肿瘤内科 Department of Medical Oncology, the First Medical Center of Chinese PLA General Hospital, Beijing 100853, China; 2 075000 张家口，解放军第八十一集团军医院肿瘤内科 Department of Medical Oncology, the Hospital of 81^st^ Group Army PLA, Zhangjiakou 075000, China; 3 100853 北京，解放军总医院第一医学中心肿瘤内科实验室 Key Laboratory of Cancer Center, the First Medical Center of Chinese PLA General Hospital, Beijing 100853, China

**Keywords:** *EGFR*突变, PD-1/PD-L1抑制剂, 肺肿瘤, PD-L1, *EGFR* mutation, PD-1/PD-L1 inhibitors, Lung neoplasms, PD-L1

## Abstract

近年来，有关程序性死亡受体1（programmed death-1, PD-1）及其配体（programmed death-1 ligand, PD-L1）抑制剂的研究取得突破性进展，迅速改变着非小细胞肺癌（non-small cell lung cancer, NSCLC）的治疗模式。但表皮生长因子受体（epidermal growth factor receptor, *EGFR*）突变患者应用PD-1/PD-L1抑制剂的治疗效果并不理想。既往研究显示，肿瘤细胞PD-L1表达率与免疫抑制剂治疗效果存在相关性。但目前*EGFR*突变对PD-L1表达的影响并不能达成一致。我们将对相关研究进行总结，以期对基础研究或临床治疗有所帮助。

## Abstract (en)

Researches on the inhibitors of programmed death-1 (PD-1) and programmed death-1 ligand (PD-L1) enjoy considerable breakthroughs in recent years, which has brought relative changes in the therapeutic model of non-small cell lung cancer (NSCLC) at an unexpected speed. However, it seems that PD-1/PD-L1 inhibitors are less effective in patients with epidermal growth factor receptor (*EGFR*) mutation than those without. Previous studies have shown that the expression rate of PD-L1 on tumor cells is correlated with the therapeutic effect of PD-1/PD-L1 inhibitors. Yet, there is no complete agreement on the effect of *EGFR* mutation on PD-L1 expression. In this review, relevant studies will be summarized with an expectation of making some contributions to basic researches and to the clinical treatment.

肺癌，尤其是非小细胞肺癌（non-small cell lung cancer, NSCLC），是肿瘤相关性死亡的最主要原因^[1]^。在传统手术治疗、铂类为基础的化疗以及放疗快速发展的背景下，NSCLC的治疗效果仍不能达到理想状态。目前，以吉非替尼、厄洛替尼、奥希替尼等为代表的表皮生长因子受体酪氨酸激酶抑制剂（epidermal growth factor receptor tyrosine kinase inhibitors, EGFR-TKIs）成为存在*EGFR*突变NSCLC患者的标准一线治疗方案^[2]^，其客观缓解率可达到70%左右甚至更高，但通常1年左右就会产生耐药^[3]^。因此，亟待寻求新的治疗方法以应对EGFR突变的NSCLC患者耐药的发生。近年来，以程序性死亡受体1（programmed death-1, PD-1）及其配体（programmed death-1 ligand, PD-L1）抑制剂为代表的免疫检查点抑制剂（immune checkpoint inhibitors, ICIs）在肺癌治疗中取得突破性进展，但既往研究^[4-7]^却显示其对*EGFR*突变患者的治疗效果并不理想。这就说明，*EGFR*突变很可能通过影响肿瘤免疫微环境，而进一步影响PD-1/PD-L1抑制剂对肿瘤的杀伤效果。PD-L1表达率是评估PD-1/PD-L1抑制剂治疗效果的潜在因素，尤其是其高表达的患者可以从相关治疗中获益更多^[8]^。我们将进一步总结*EGFR*突变对PD-L1表达影响的基础及临床研究，并对其结果存在差异的原因进行简单探讨，希望可以对相关研究及PD-1/PD-L1抑制剂的临床应用提供有效的建议及指导。

## 1 *EGFR*突变导致PD-L1表达上调相关研究

### 1.1 基础研究

2014年，Azuma团队^[9]^对14种人NSCLC细胞系（PC9、HCC827、NCI-H1975、QG56、1-87、H1299、H2122、H322、H460、LK2、LK87、H23、A549、H157）进行流式细胞分析技术检测发现存在*EGFR*突变的细胞系PD-L1表达明显高于EGFR野生型细胞系（*P*=0.023），且*EGFR*突变型细胞系HCC827、PC-9、H1975可以检测到EGFR高水平的磷酸化作用（表 1）。为了探索EGFR通路对PD-L1表达的影响，研究者进一步对*EGFR*突变细胞系HCC827、PC-9应用厄洛替尼处理后发现其PD-L1表达下调以及EGFR磷酸化受到抑制，但对EGFR野生型细胞系A549、H1975应用厄洛替尼处理后并未影响其PD-L1的表达及EGFR磷酸化水平。该研究说明，*EGFR*突变可以导致人NSCLC细胞系PD-L1表达上调。

**1:** 依据基因突变状态和组织病理学分类的肺癌细胞系 Lung cancer cell lines classified according to oncogene status and histology

Cell line	*EGFR* status	Histology
PC-9	Del (E746-A750)	Adenocarcinoma
HCC827	Del (E746-A750)	Adenocarcinoma
H1650	Del (E746-A750)	Adenocarcinoma
11-18	L858R	Adenocarcinoma
H1975	L858R+T790M	Adenocarcinoma
A549	Wild type	Adenocarcinoma
H1437	Wild type	Adenocarcinoma
H1573	Wild type	Adenocarcinoma
H1993	Wild type	Adenocarcinoma
H1944	Wild type	Adenocarcinoma
H23	Wild type	Adenocarcinoma
H2122	Wild type	Adenocarcinoma
H322	Wild type	Adenocarcinoma
LK87	Wild type	Adenocarcinoma
SPC-A1	Wild type	Adenocarcinoma
1-87	Wild type	Adenocarcinoma
H1299	Wild type	Non-small cell lung cancer
QG56	Wild type	Non-small cell lung cancer
H460	Wild type	Large cell
LU-99	Wild type	Large cell
LK2	Wild type	Squamous
H157	Wild type	Squamous
H596	Wild type	Adenosquamous
Beas-2B	Wild type	Bronchiolar epithelial cell
EGFR: epidermal growth factor receptor.		

Chen等^[10]^于2015年对人NSCLC细胞系A549、PC-9、HCC827、H1975、H1993以及Beas-2B细胞系进行的相关研究进行了报道。该研究主要由四部分组成：①研究者通过蛋白质印迹法、RT-PCR检测发现，*EGFR*突变NSCLC细胞系（PC-9、HCC827、H1975）PD-L1表达水平明显高于EGFR野生型细胞系（A549、H1993）和Beas-2B细胞系；并进一步应用免疫组织化学染色法及流式细胞分析技术对H1975和A549细胞系PD-L1表达情况进行验证，结果与之前一致。②研究者使用了EGF去激活EGFR野生型细胞系，研究发现随着EGF激活剂量的升高（0 ng/mL-40 ng/mL）Beas-2B细胞系中p-EGFR和PD-L1蛋白表达也逐渐升高；随后，研究者对该细胞系进行了转染来促进不同强度的EGFR-19del以及EGFR-L858R表达，发现同样可以导致p-EGFR和PD-L1表达水平的升高。③研究者发现吉非替尼可以逆转EGF激活导致的p-EGFR以及PD-L1表达上调；可以逆转Beas-2B-EGFR-L858R细胞系PD-L1表达上调；可以降低PC-9、HCC827细胞系的PD-L1蛋白表达；吉非替尼不会使H1975细胞系p-EGFR以及PD-L1表达发生变化，但C0-1686（三代EGFR-TKI）可以使该细胞系PD-L1表达下调。④研究者进一步研究认为EGFR突变是通过p-ERK 1/2/p-c-jun信号通路而不是p-AKT/p-S6通路影响PD-L1的表达。该研究团队进一步验证了Azuma团队的相关研究，不仅说明了EGFR突变可以导致人NSCLC细胞系PD-L1表达上调，并对EGFR影响PD-L1表达的通路机制进行了简单探索。

随后，Lin等^[11]^通过流式细胞分析技术发现*EGFR*突变细胞系（PC-9、HCC827、NCI-H1650）比EGFR野生型细胞系（NCI-H1299、NCI-H460、SPC-A1）PD-L1表达高。同样，为了确定EGFR的激活可以诱导PD-L1表达，研究者应用了重组人EGF（100 ng/mL）刺激H460细胞系，24 h后在该细胞系中发现了升高的PD-L1 mRNA及蛋白质。充分说明EGFR激活可导致PD-L1表达上调。研究者进一步对PC-9以及HCC827细胞系进行不同剂量吉非替尼培养48 h后发现，其PD-L1表达水平下调并且呈剂量依赖型。对严重免疫缺陷小鼠移植PC-9细胞系后应用吉非替尼并与溶媒组进行对比，发现应用吉非替尼的小鼠PD-L1表达低于对照组。相较之前的研究，该研究进一步说明应用EGFR-TKIs可以降低小鼠体内PC-9细胞系的PD-L1表达。

Ota研究团队^[12]^在2015年通过RT-PCR及流式细胞分析技术对*EGFR*突变细胞系（HCC827、H1975、PC-9、11-18、H1650）及野生型细胞系（H322、A549、1-87、LK87、H23、H2122、H1437、H1573、H1944、H157、H596、H460、H1299）的PD-L1 mRNA以及PD-L1细胞膜蛋白表达进行检测。发现*EGFR*突变型细胞系PD-L1 mRNA以及细胞膜蛋白表达均高于野生型。并且进一步发现，应用厄洛替尼可以使HCC827细胞系PD-L1 mRNA以及PD-L1细胞膜蛋白表达下调。

2016年Zhang等^[13]^同样采用RT-PCR和蛋白印迹分析技术对NSCLC细胞株EGFR野生型（A549、H1299、SPC-A1）和突变型（PC-9、H1975、HCC827）细胞系进行研究，发现*EGFR*突变细胞系PD-L1表达高于野生型。但H1299与PC-9细胞系PD-L1表达相近，考虑与PC-9细胞系TP53缺失有关，并且已有研究^[14]^证实其与PD-L1表达水平具有相关性，这也就说明共突变的存在可以进一步影响PD-L1的表达水平。同时该研究团队进一步发现，*EGFR*突变影响PD-L1表达的通路可能为IL-6/JAK/STAT3。

2019年Guo研究团队^[15]^对人NSCLC细胞系H522、H661、HCC827、H1299、HCC2935、H1650、H1792、H1975进行蛋白印迹技术检测发现，三种*EGFR*突变细胞系（HCC827、HCC2935、H1975）PD-L1表达水平高于EGFR野生型细胞系（H522、H661、H1792、H1299）。为了进一步探究*EGFR*突变对PD-L1表达的影响，研究者对H661细胞系进行转染（e19del、e19del+T790M、L858R、L858R+T790M），发现经转染的细胞系PD-L1表达高于EGFR野生型细胞系。该研究较其他研究进一步发现了*EGFR*突变细胞系较野生型细胞系IκBα及低氧诱导因子-1α（hypoxia inducible factor-1α, HIF-1α）升高。

在进行*EGFR*突变对PD-L1表达影响的基础研究过程中，Abdelhamed团队^[16]^、Lastwika团队^[17]^的研究结果也显示了*EGFR*突变可以上调PD-L1表达，并分别对相关影响通路AKT/STAT3以及AKT/mTOM进行了进一步的简要探索。

### 1.2 临床研究

见表 2。Azuma团队^[9]^在体外细胞系研究的基础上进一步对164例NSCLC患者进行PD-L1表达检测发现，*EGFR*突变患者PD-L1表达率明显高于EGFR野生型（*P* < 0.001），且*EGFR*突变是导致PD-L1表达上调的独立因素（OR=25.4, 95%CI: 2.9-47.9, *P*=0.027）。

**2:** 已发表NSCLC患者*EGFR*突变状态与PD-L1表达相关性的研究汇总 Published report that have studied the relationship of *EGFR* mutation and PD-L1 expression in NSCLC

Author (Year)	Ethnicity (Country)	PD-L1 Ab	Patient number (*EGFR* mutation)	Ad/Sq/other	Ⅰ/Ⅱ/Ⅲ/Ⅳ	Method	Cut-off	Influence
Azuma^[9]^ (2014)	Asian (Japan)	Lifespan Bioscience	164 (57)	114/50	67/46/51/0	IHC	Intensity×extent	Up
D’Incecco^[18]^ (2015)	Caucasian (Italian)	Ab58810	125 (56)	83/23/19	0/0/0/125	IHC	≥5%	Up
Tang^[19]^ (2015)	Asian (China)	E1L3N	170 (99)	Ad 145 Non-Ad 25	0/0/9/161	IHC	H-score≥5%	Up
Song^[20]^ (2016)	Asian (China)	Proteintech	385 (205)	385/0/0	121/79/185/0	IHC	≥5%	Up
Huynh^[21]^ (2016)	Caucasian (America)	E1L3N	261 (54)	261/0/0	201/34/22/4	IHC	≥1%；≥5%；≥50%	Down
Ji^[22]^ (2016)	Asian (China)	Ab174838	100 (60)	100/0/0	42/27/41	IHC	> 5% 2+ (moderate)	Down
Inoue^[23]^ (2016)	Asian (Japan)	E1L3N	654 (132）	430/179/45	416/113/125	IHC	H-score≥5	Down
Dong^[24]^ (2017)	Asian (China)	SP142	255 (Unclear)	Unclear	Unclear	IHC	< 5%；5%-49%；≥50%	Down
Takada^[25]^ (2017)	Asian (Japan)	SP142	499 (112)	417/82/0	354/82/63	IHC	1%; 5%; 10%; 50%	Down
Takada^[26]^ (2018)	Asian (Japan)	SP142	441 (223)	441/0/0	339/54/40/8	IHC	0%; 1%-4%; 5%-49%; ≥50%	Down
Lee^[28]^ (2019)	Asian (Korea)	22C3	1, 000 (424）	785/188/27	621/167/140/30	IHC	< 1%; 1%-49%; ≥50%	Down
Yang^[29]^ (2014)	Asian (Taiwan)	Proteintech	163 (97)	163/0/0	163/0/0/0	IHC	≥5%	No
Yang^[30]^ (2016)	Asian (Taiwan)	Proteintech	105 (17)	0/105/0	105/0/0/0	IHC	≥5%	No
Zhang^[31]^ (2014)	Asian (China)	SAB 2900365	143 (76)	143/0/0	Ⅰ: 66; Ⅱ-Ⅲ: 77	IHC	Quickscore (range 0-18)	No
Cooper^[32]^ (2015)	Caucasian (Australian)	22C3	678 (33)	276/271/131	49/271/315	IHC	≥50% (any intensity)	No
Kim^[33]^ (2015)	Asian (Korean)	E1L3N	331 (7)	0/331/0	131/118/79	IHC	≥10% (any intensity)	No
Schmidt^[34]^ (2015)	Caucasian (German)	E1L3N	321 (6)	125/149/47	187/83/51	IHC	≥5% 2+ (moderate)	No
Gainor^[35]^ (2016)	Caucasian (America)	E1L3N	95 (68)	48/9/1	Unclear	IHC	≥1%; ≥5%; ≥50% (any intensity)	No
Ameratunga^[36]^(2016)	Caucasian (Australian)	E1L3N	522 (27)	288/182/57	Unclear	IHC	> 5% 2+ (moderate)	No
PD-L1: programmed death ligand 1; EGFR: epidermal growth factor receptor; NSCLC: non-small cell lung carcinoma; Ad: adenocarcinoma; Sq: squamous cell carcinoma.								

D’incecco等^[18]^对125例晚期NSCLC患者进行PD-L1表达分析，其中包括56例*EGFR*突变、29例*KRAS*突变、10例*ALK*突变、30例EGFR/KRAS/ALK野生型。在进行分析的标本中，有78.4%的标本来自于原发病灶，13.6%的标本来自于转移病灶。其中123例标本成功进行了PD-L1表达评估，中位表达水平为75。对29例三阴性患者标本进行评估后发现中位PD-L1表达水平是20，明显低于*EGFR*突变120（*P* < 0.001）、*ALK*突变115（*P*=0.02）、*KRAS*突变55（*P*=0.06）。在*EGFR*突变标本中PD-L1阳性率较高，具有统计学差异（*P* < 0.001）。

Tang等^[19]^研究了170例晚期NSCLC患者的PD-L1过表达率是65.9%（112/170）。其中，99例患者存在*EGFR*突变。PD-L1过表达在*EGFR*突变患者与野生型患者中分别占71.9%（64/89）、57.1%（32/56）（*P*=0.067）。

Song等^[20]^对385例肺腺癌PD-L1表达情况进行研究。该研究中，肿瘤比例评分（tumor proportion score, TPS）≥5%视为表达阳性。该研究发现205例*EGFR*突变患者中，112例（54.6%）PD-L1表达阳性；180例EGFR野生型患者中74例（41.1%）表达阳性（*P*=0.008）。研究者进一步发现在385例患者中有24例存在基因突变共表达的情况，且共突变比单基因突变对PD-L1阳性表达的影响更大（*P* < 0.001）。205例*EGFR*突变患者中包括14例共突变患者，这就说明基因共突变存在也是可能影响研究结果的因素。

## 2 *EGFR*突变导致PD-L1表达下调的临床研究

Huynh等^[21]^对261例肺腺癌进行PD-L1表达评估。该研究排除既往进行过新辅助化疗的患者。包括54例*EGFR*突变患者，有5例TPS≥5%，49例TPS < 5%。该研究发现，*EGFR*突变与PD-L1表达呈负相关（*P* < 0.001）。

Ji等^[22]^对100例原发性肺腺癌患者标本进行PD-L1表达情况检测。已排除既往行新辅助化疗或有过恶性肿瘤病史的患者。该研究将cut-off值设置为5%。60例*EGFR*突变患者中，42例PD-L1低表达，18例高表达；EGFR野生型患者中18例低表达，22例高表达。该研究发现存在*EGFR*突变的患者PD-L1表达降低（*P*=0.012）。

Inoue等^[23]^对654例NSCLC患者进行研究，包括腺癌430例（65.7%）、鳞癌179例（27.4%）、其他45例（6.9%）。其中*EGFR*突变患者132例（20.2%），PD-L1表达阳性25例（12.4%），阴性107例（23.6%）；EGFR野生型522例（79.8%），PD-L1表达阳性176例（87.6%），阴性346例（76.4%）。该研究同样发现*EGFR*突变可以下调PD-L1表达（*P*=0.001）。

Dong等^[24]^对15项研究进行了汇总分析发现存在*EGFR*突变的患者PD-L1表达较低（OR=1.79, 95%CI: 1.10-2.93, *P*=0.02）。为了对该汇总分析结果进行验证，研究者对肿瘤基因图谱研究（The Cancer Genome Atlas, TCGA）中的237例肺腺癌患者进行反向蛋白质阵列分析（reverse phase protein microarray, RPPA），发现*EGFR*突变患者的PD-L1表达蛋白要比EGFR野生型低（*P*=0.014）；并对通过全基因组测序（whole-genome sequencing, WGS）对广东省肺癌研究所（Guangdong Lung Cancer Institute, GLCI）的mRNA情况进行分析，也显示*EGFR*突变患者PD-L1表达低于EGFR野生型患者（*P*=0.044）。研究者进一步对GLCI 255例NSCLC患者分析发现，*EGFR*突变和PD-L1表达具有负相关性（*P*=0.014）。

2017年Takada研究团队^[25]^检测了499例原发NSCLC患者PD-L1表达情况，并采用了4种不同cut-off值进行评估。这些患者中排除新辅助治疗以及既往头颈部、食道鳞癌患者。*EGFR*突变在腺癌患者中检测，其中有效标本为235例，包括112例（47.7%）突变型以及123例野生型（52.3%）。按照1%、5%、10%、50% cut-off值分别进行分类的情况下，*EGFR*突变肺腺癌患者的PD-L1表达均低于EGFR野生型患者。于2018年，该研究团队^[26]^再次对441例原发肺腺癌PD-L1表达情况进行研究发现，EGFR野生型与PD-L1的阳性表达具有相关性（*P* < 0.000, 1）。同时，该研究进一步发现*EGFR*不同位点的突变与PD-L1表达无明显相关性（*P*=0.599, 9），但PD-L1 TPS 5%-49%在EGFR 19del患者中明显高于EGFR外显子21 L858R突变（12.2% *vs* 2.6%）。

Li等^[27]^分析了21项研究的4, 857例患者，1, 435例存在*EGFR*突变的患者中608例（36.7%）存在PD-L1阳性表达；3, 422例突变野生型患者中1, 456例（44.1%）存在PD-L1阳性表达。PD-L1表达与EGFR野生型存在相关性（OR=0.68, 95%CI: 0.48-0.96, *P*=0.03）。

近期，Lee等^[28]^对1, 000例手术切除NSCLC患者（腺癌785例，鳞癌188例，21例大细胞神经内分泌癌，4例类癌，2例小细胞肺癌）PD-L1表达情况进行了研究。在按照 < 1%、1%-49%、≥50% cut-off值分别进行分类的情况下，发现肺腺癌中存在*EGFR*突变患者（424例）的PD-L1表达率明显低于EGFR野生型患者（*P* < 0.000, 1）。

## 3 *EGFR*突变导致PD-L1表达改变无统计学意义

2014年Yang等^[29]^对163例肺腺癌患者PD-L1表达情况进行研究。PD-L1 TPS≥5%视为表达阳性。结果显示，PD-L1阳性表达率为39.9%（65/163），但其阳性表达水平与常见的肺腺癌突变基因*EGFR*（*P*=0.193）、*KRAS*（*P*=0.268）、*BRAF*（*P*=0.438）和*ALK*（*P*=0.564）均没有相关性。2016年该团队又对105例肺鳞癌患者PD-L1表达情况进行研究^[30]^。同样，PD-L1 TPS≥5%视为表达阳性。PD-L1阳性表达率为56.2%（59/105），且阳性表达水平与突变基因*EGFR*（*P*=0.561）、*KRAS*（*P*=0.255）、*BRAF*（*P*=0.064）和*ALK*（N/A）均不具有相关性。

Zhang^[31]^对143例肺腺癌PD-L1表达情况进行分析。并排除既往行新辅助化疗或有过恶性肿瘤病史的患者。76例*EGFR*突变患者中，37例PD-L1表达阳性，39例表达阴性，差异无统计学意义（*P*=0.946）。

Cooper研究团队^[32]^检测了678例NSCLC患者PD-L1表达情况。其中包括腺癌276例、鳞癌271例、大细胞癌116例、混合3例、其他病理类型12例。肿瘤细胞TPS显示≥50%或H-Score≥50考虑为PD-L1高表达。*EGFR*突变患者两种分级方法中不同PD-L1表达情况患者的数量均相同。EGFR野生型且PD-L1 TPS < 50%的患者222例（93.7%），PD-L1 TPS≥50%的患者15例（6.3%）；*EGFR*突变型PD-L1 TPS < 50%的患者33例（100%），差异无统计学意义（*P*=0.23）。

Kim^[33]^、Schmidt^[34]^、Gainor^[35]^、Ameratunga^[36]^等团队，在相关研究的过程中也发现NSCLC患者PD-L1表达情况与*EGFR*基因状态无明显相关性。

## 4 研究结果不一致原因分析

在对既往相关研究进一步学习的过程中，我们不难发现，*EGFR*突变对PD-L1表达相关影响的研究结果存在不一致性，我们总结出以下几点可能的原因：①大多数临床研究为回顾性研究，存在选择偏倚，且纳入患者数量不同，有些研究纳入患者数量较少。②在这些研究中，患者基线特征的异质性也会影响研究的结果，比如患者不同地域的分布、病理分型及分期。有相关研究显示，亚裔*EGFR*突变概率要高于非亚裔，在亚洲人群中*EGFR*突变在NSCLC中占39.6%，在肺腺癌中更是高达50%^[37]^；鳞癌与腺癌基因突变的概率与种类不同^[38]^；PD-L1阳性表达在鳞癌NSCLC中比腺癌更常见^[39, 40]^。③免疫治疗领域目前缺乏关于PD-L1标准化测试，许多不同的评分标准与试剂被应用^[41]^。有研究^[42]^发现对同一标本应用不同抗体PD-L1蛋白表达结果不同；另外有研究发现，应用SP142染色的PD-L1表达阳性率低于其他抗体，如：28-8、22C3和SP263^[43]^。④PD-L1阳性表达的临界值不同。⑤依据既往研究传统化疗^[44]^、抗血管治疗^[45-47]^、EGFR-TKIs^[10, 11]^均会对PD-L1表达有影响，本文提及的临床研究部分未明确描述进行PD-L1检测前既往治疗情况。

## 5 总结与展望

随着PD-1/PD-L1抑制剂治疗NSCLC相关研究的不断进展，肿瘤突变驱动基因对其治疗疗效的影响随之被关注。肿瘤微环境是肿瘤细胞赖以生存和发展的复杂环境，肿瘤细胞PD-L1表达情况与PD-1/PD-L1免疫抑制剂治疗效果存在一定相关性。因此，EGFR通路对PD-L1表达的影响也被进一步探索。虽然目前相关研究结果存在差异，但我们期待在未来研究的过程中可以对*EGFR*突变影响PD-L1表达水平的多条信号通路进行探索，并完成多中心、随机性、前瞻性的临床研究。希望在进一步的探索过程中，可以为*EGFR*突变患者找到提高PD-1/PD-L1抑制剂治疗疗效的突破点。
